# Combination Effects of *Clostridium butyricum* and Glucose Oxidase on Intestinal Antioxidant Status, Immune Responses, Barrier-Related Gene Expression, and Cecal Short-Chain Fatty Acid Profiles in Broilers

**DOI:** 10.3390/biology15171560

**Published:** 2026-09-07

**Authors:** Jie Ma, Feilong Liu, Xinhao Zhang, Henghuan Chen, Yan Yan, Guodong Lei, Hengbo Liu, Aiqin Gao

**Affiliations:** College of Animal Science, Inner Mongolia Agricultural University, Hohhot 010018, China; 18195167236@163.com (J.M.);

**Keywords:** *Clostridium butyricum*, glucose oxidase, broilers, intestinal health

## Abstract

The potential of combining *Clostridium butyricum* (CB) and glucose oxidase (GOD) in poultry production remains to be systematically evaluated. This study evaluated the effects of dietary CB and GOD, supplied alone or in combination, on ileal antioxidant status, jejunal immune responses, gut epithelial barrier function, and cecal short-chain fatty acid (SCFA) composition in broilers, with the aim of comparing their practical effects rather than investigating synergistic or interactive effects. A total of 192 1-day-old broilers (white-feathered) were randomly distributed among 4 experimental groups, each having 6 replicate pens of 8 chicks. The groups had comparable starting weights (43.53 ± 0.51 g) and an equal male-to-female ratio. The control diet (CON) contained no additives, whereas the other three diets were formulated with CB at an inclusion rate of 400 mg/kg, GOD at the same level, or a mixture of both (400 mg/kg each), corresponding to the CB, GOD, and MIX groups, respectively. This experiment spanned 42 days, comprising an early stage (from day 1 to day 21) and a later stage (from day 22 to day 42). At day 21, the GOD group exhibited a significant elevation in ileal SOD activity relative to the CON group (*p* < 0.05). Jejunal interleukin-6 (IL-6) levels were significantly decreased in the CB and MIX groups (*p* < 0.05), while the MIX group also showed a significant decrease in IL-2 and a significant increase in interleukin-10 (IL-10) levels (*p* < 0.05). The GOD and MIX groups showed a significant elevation of sIgA (*p* < 0.05), while the CB and GOD groups exhibited marked upregulation of *Occludin* mRNA (*p* < 0.05). Butyrate concentrations in the cecum were significantly higher in the CB group than in the CON group (*p* < 0.05). Likewise, acetate levels were elevated in the MIX group compared with CON (*p* < 0.05). At day 42, the MIX group exhibited a significant reduction in ileal MDA content compared with the control (*p* < 0.05). In the jejunum of the MIX group, IL-6 was significantly reduced and IL-10 was significantly elevated (*p* < 0.05). Additionally, *ZO-1* mRNA expression was markedly upregulated in the same group (*p* < 0.05). Ileal GSH-Px activity was significantly higher only in the CB group (*p* < 0.05). Overall, combined supplementation with CB and GOD exerted beneficial effects on broiler antioxidant status, immune responses, intestinal barrier-related parameters, and cecal SCFA composition, although the magnitude of these effects varied among endpoints and sampling ages.

## Simple Summary

The modern livestock and poultry industry has an urgent need for nontoxic alternatives to antibiotics of natural origin, thereby safeguarding animal health. It was revealed by this study that co-supplementation with *Clostridium butyricum* (CB) and glucose oxidase (GOD) exerted a beneficial effect on the intestinal health of broilers. According to the results, the CB and GOD combination exerted combined effects across multiple dimensions—including antioxidant capacity, immune function, gut barrier integrity, and cecal short-chain fatty acid profiles—at different growth stages. These findings indicate that this enzyme–probiotic combination holds considerable promise as a safe and natural antibiotic alternative in broiler diets, helping to reduce antibiotic dependence and promote more sustainable poultry production.

## 1. Introduction

A healthy gastrointestinal tract is essential for poultry production because it facilitates efficient nutrient utilization, sustains growth performance, and protects against pathogen invasion [1]. With the increasing scale and intensification of livestock and poultry production, high-density rearing practices can readily impair intestinal health [2]. Historically, antibiotics were widely used as feed additives to enhance growth performance [3]. With the global restriction of antibiotic growth promoters (AGPs), the development of green feed additives has become a research priority [4]. The poultry industry has extensively utilized feed enzymes and probiotics as typical examples of eco-friendly feed additives [5,6]. In particular, *Clostridium butyricum* (CB), a butyrate-producing probiotic, and glucose oxidase (GOD), an enzyme that improves the intestinal redox environment, have shown beneficial effects individually.

A substantial body of research indicates that probiotics can suppress pathogenic bacteria, boost oxidative defense, reduce toxin-induced damage, and support intestinal well-being in poultry [7,8,9]. CB is now recognized as an efficacious probiotic that can enhance growth performance and preserve intestinal barrier integrity in animals [10,11]. CB is a Gram-positive, anaerobic, spore-forming bacterium belonging to the genus *Clostridium*. It has been reported to positively influence intestinal immune status, preserve epithelial barrier integrity, and mitigate inflammatory damage [12,13]. CB, similar to *Bacillus* species, is capable of spore formation, endowing it with tolerance to heat, gastric acid, and bile salts, properties that are critical for its use in feed [14,15].

Feed enzymes, which represent a major category of green feed additives, are mainly derived from bacterial and fungal microorganisms and have been widely applied in animal nutrition [16]. GOD belongs to the family of aerobic dehydrogenases. The enzyme drives the oxidation of β-D-glucose to gluconic acid (GA), with simultaneous H_2_O_2_ formation and substantial oxygen utilization [17]. Given these characteristics, GOD possesses considerable market value across food processing, clinical diagnostics, oral hygiene products, chemical manufacturing, and biotechnological applications [18]. Critically, GOD exerts antioxidative effects and reduces oxidative injury. In parallel, it regulates intestinal pH, restricts opportunistic pathogens, and fortifies the gut mucosal barrier. These actions collectively contribute to superior growth rates and enhanced immunological status in poultry and livestock [19,20,21,22]. Consequently, GOD is recognized as an effective antibiotic alternative and a promising novel feed additive for animal production. The GA can be utilized by certain intestinal microorganisms and may contribute to short-chain fatty acid (SCFA) production, depending on microbial community composition and metabolic cross-feeding [23].

Although the individual effects of CB and GOD in broilers are well documented, their combined use remains poorly understood. We hypothesized that combined supplementation would produce responses different from those observed with either additive alone or the unsupplemented control. This study therefore aimed to compare the practical effects of CB alone, GOD alone, and their combination, rather than to investigate synergistic or interactive effects, and to provide baseline data for the rational combined use of these additives in poultry diets.

## 2. Materials and Methods

All animal care and use protocols in this work were reviewed and approved by the Laboratory Animal Welfare and Ethics Committee of Inner Mongolia Agricultural University (approval number NND2026083).

### 2.1. Animals and Experimental Design

A total of 192 1-day-old Ross 308 broilers (white-feathered) were randomly distributed among 4 experimental groups, each having 6 replicate pens of 8 chicks. The groups had comparable starting weights (43.53 ± 0.51 g) and an equal male-to-female ratio. The sex ratio was balanced across treatments but was not included as a separate factor in the statistical model because the primary aim was to evaluate treatment effects. For all physiological and molecular measurements, the pen (replicate) served as the experimental unit; individual bird values were averaged within each pen before statistical analysis, so that *n* = 6 pens per treatment group. The objective of this work was to assess the effects of dietary CB and GOD supplementation, whether used singly or in combination. CB and GOD were provided by Wuhan Sunhy Biological Co., Ltd. (Wuhan, China). The CB product contained ≥ 1 × 10^10^ CFU/g and was supplemented at 400 mg/kg, corresponding to a theoretical viable dose of 4 × 10^12^ CFU/kg diet. The GOD product had an enzyme activity of ≥4000 U/g and was supplemented at 400 mg/kg, corresponding to 1.6 × 10^6^ U/kg diet (calculated using the minimum guaranteed activity). The four dietary treatments consisted of a basal diet without any supplement (CON) and the basal diet amended with 400 mg/kg CB, 400 mg/kg GOD, or both additives at the same dosage (400 mg/kg each), which were designated as the CB, GOD, and MIX groups, respectively. Both additives were uniformly blended with the basal diets at the specified concentrations. The dietary formulation for the basal diet followed the specifications set forth in the *Chinese Chicken Feeding Standard* (NY/T33-2004) [24]. Shown in Table 1 are the basal diet’s ingredient composition and nutrient levels. This feeding trial was conducted at a commercial broiler production farm. Temperature and humidity were controlled as described by Ma et al. [25]: the ambient temperature was maintained at 33 °C during the first week and then lowered by 3 °C per week until reaching 21 °C, while the relative humidity was kept at approximately 65%. The 42-day trial comprised two distinct feeding phases: a starter period from day 1 to day 21, followed by a grower period from day 22 to day 42. During the 42-day trial, broilers were maintained on free access to feed and water and were given routine immunizations as per the normal schedule.

### 2.2. Sample Collection

At day 21 and day 42, following a 12 h fast, we measured individual body weights and randomly selected one bird from each replicate pen. The selected birds were euthanized by exsanguination under anesthesia. Tissues were then promptly harvested: ileal and jejunal segments, jejunal mucosa (gently scraped with a scalpel), and cecal digesta. All specimens were snap-frozen in liquid nitrogen and subsequently stored at −80 °C for later analysis.

### 2.3. Intestinal Antioxidant and Immune Index Assays

We processed 0.5 g each of ileal and jejunal mucosal samples in 4.5 mL of normal saline to yield a 1:9 (*w*/*v*) homogenate. The homogenates were then spun at 3500 rpm for 15 min, and the supernatants were harvested and stored for later analysis. For ileal antioxidant assessment, we used kits (Nanjing Jiancheng Bioengineering Institute, Nanjing, China) to measure total superoxide dismutase (T-SOD), total antioxidant capacity (T-AOC), malondialdehyde (MDA), catalase (CAT), and glutathione peroxidase (GSH-Px). Tissue protein concentration was also determined using a kit from the same supplier (Nanjing Jiancheng Bioengineering Institute, Nanjing, China). Jejunal immune parameters—interleukin-1β (IL-1β), interleukin-2 (IL-2), interleukin-6 (IL-6), interleukin-10 (IL-10), tumor necrosis factor-α (TNF-α), and secretory immunoglobulin A (sIgA)—were quantified with ELISA kits from the same supplier, with all procedures conducted according to the manufacturer’s protocols.

### 2.4. Real-Time QPCR

Total RNA was extracted from 50 mg of jejunal mucosa using the TaKaRa RNAiso Plus kit (Takara Bio, Beijing, China) after homogenization with an FA 25D homogenizer (Fluko Technology Development, Shanghai, China) according to Ji et al. [26]. RNA purity and concentration were assessed using a NanoDrop spectrophotometer, (Thermo Fisher Scientific, Wilmington, DE, USA) with A260/A280 ratios between 1.8 and 2.0 considered acceptable. RNA integrity was verified by 1.5% agarose gel electrophoresis with clear 28S and 18S rRNA bands. For cDNA synthesis, the Evo M-MLV RT kit (Accurate Biotechnology, Changsha, China) was used: 2 μL of RNA (500 ng/μL), 2 μL of genomic DNA removal reagent, and 6 μL of water were incubated at 42 °C for 2 min in a LabCycler (SensoQuest, Göttingen, Germany), followed by addition of 4 μL of 5× reaction mix and 6 μL of water, with the reaction run at 37 °C for 15 min, 85 °C for 5 s, and 4 °C hold. Real-time PCR was performed on a LightCycler 480 II system (Roche, Basel, Switzerland) using the SYBR Green Pro Taq HS qPCR Kit (Accurate Biotechnology). Each 20 μL reaction contained 2 μL of cDNA, 0.5 μL of each primer, 10 μL of 2× SYBR Green Premix, and 7.2 μL of RNase-free water. The cycling protocol consisted of 95 °C for 30 s, followed by 40 cycles of 95 °C for 5 s and 60 °C for 30 s. Melt-curve analysis was performed after amplification to confirm the specificity of each primer pair, and a single peak was observed for all target genes. Amplification efficiencies were determined from standard curves generated by serial dilution of pooled cDNA and ranged from 90% to 110% for all primer pairs. β-Actin was selected as the internal control based on its stable expression across dietary treatments and sampling time points in preliminary experiments; its cycle threshold values were not significantly affected by treatment or age in the present study. Relative expression of *Occludin*, *ZO-1*, *JAM2*, and *MUC2* was calculated using the 2^−ΔΔCt^ method [27]. Primers were designed and checked in silico using NCBI Primer-BLAST, and their sequences are listed in Table 2.

### 2.5. Cecal SCFAs Analysis

SCFA analysis was performed using a Shimadzu GC-2014 gas chromatograph equipped with a flame ionization detector (FID) and a DB-FFAP capillary column (30 m × 0.25 mm × 0.25 μm; Agilent J&W, Santa Clara, CA, USA). Hydrogen was used as the carrier gas at a constant flow rate of 1.5 mL/min. The injector and detector temperatures were 170 °C and 250 °C, respectively. Samples were injected in split mode at a split ratio of 10:1 with an injection volume of 1 μL. The oven temperature program followed Xiao et al. [28]: initial temperature of 70 °C, increased to 180 °C at 15 °C/min (held for 3 min), then increased to 250 °C at 40 °C/min (held for 5 min). Individual SCFAs were identified by comparing their retention times with those of authentic standards, including acetic, propionic, butyric, isobutyric, valeric, and isovaleric acids. Quantification was performed using the internal standard method with 2-ethylbutyric acid (2-EB) as the internal standard. A mixed standard solution containing the six SCFAs at concentrations of 0.1, 0.5, 1, 5, 10, and 20 mM was used to generate calibration curves, and the linear range was confirmed. SCFA concentrations in cecal digesta were expressed as μmol/g of fresh digesta.

Prior to GC analysis, an internal standard solution was prepared by dissolving 25 g of metaphosphoric acid and 0.217 mL of 2-EB in ultrapure water to a final volume of 100 mL and stored at 4 °C. Cecal digesta (0.5 g) were thawed, weighed into 5-mL tubes, mixed with 1.25 mL of water, vortexed, and centrifuged at 12,000 rpm for 10 min at 4 °C. The supernatant was collected, mixed with the internal standard, kept on ice for 30 min, and centrifuged again at 12,000 rpm for 15 min at 4 °C. The final extract was filtered through a 0.22-μm membrane before GC injection.

### 2.6. Data Analysis

Normality and homoscedasticity of the data were assessed using the Shapiro–Wilk test and Levene’s test, respectively, with *p* > 0.05 considered to indicate that the assumptions were met [29]. Subsequently, one-way ANOVA was used to detect overall differences among the four groups, and the Tukey–Kramer post hoc test was applied for multiple pairwise comparisons. All statistical analyses were performed using IBM SPSS Statistics 26.0 (Armonk, NY, USA), with statistical significance set at *p* < 0.05.

## 3. Results

### 3.1. Effects of Dietary Supplementation with CB and GOD on Ileal Antioxidant Capacity in Broilers

The effects of CB and GOD on the ileal antioxidant capacity in broilers are presented in Table 3. On day 21, significant treatment effects were limited to elevated ileal SOD in the GOD group versus CON (*p* < 0.05); CAT, MDA, T-AOC, and GSH-Px showed no significant intergroup differences (*p* > 0.05). By day 42, the MIX group exhibited lower ileal MDA (*p* < 0.05), and the CB group displayed higher GSH-Px activity (*p* < 0.05). Across all treatment groups, T-AOC, CAT and SOD activities remained statistically unchanged (*p* > 0.05).

### 3.2. Effects of Dietary Supplementation with CB and GOD on Jejunal Immune Function in Broilers

The effects of CB and GOD on jejunal immune function in broilers are presented in Table 4. At day 21, IL-6 levels were significantly reduced in the CB and MIX groups relative to CON (*p* < 0.05). The MIX group also showed decreased IL-2 and increased IL-10 (*p* < 0.05), while sIgA was elevated in both the GOD and MIX groups (*p* < 0.05). No significant intergroup differences were found for IL-1β or TNF-α (*p* > 0.05). At day 42, the MIX group again exhibited lower IL-6 and higher IL-10 compared with CON (*p* < 0.05), whereas IL-2, IL-1β, TNF-α, and sIgA remained unchanged across treatments (*p* > 0.05).

### 3.3. Effects of Dietary Supplementation with CB and GOD on Intestinal Barrier Function in Broilers

The effects of CB and GOD on the mRNA expression of intestinal barrier-associated genes in broilers are presented in Table 5. At day 21, *Occludin* transcript levels in the jejunum were significantly higher in the CB and GOD groups than in the CON group (*p* < 0.05), whereas *JAM2*, *MUC2*, and *ZO-1* showed no significant intergroup differences (*p* > 0.05). At day 42, *ZO-1* mRNA was significantly upregulated in the MIX group relative to CON (*p* < 0.05), while *JAM2*, *MUC2* and *Occludin* expression remained comparable across all treatments (*p* > 0.05). These transcriptional changes may indicate potential regulation of barrier-associated pathways, but they do not directly demonstrate improved intestinal barrier function or permeability.

### 3.4. Effects of Dietary Supplementation with CB and GOD on Cecal SCFAs Profiles in Broilers

The effects of CB and GOD on cecal SCFAs levels in broilers are presented in Table 6. At day 21, cecal butyrate content was significantly higher in the CB group than in the CON group (*p* < 0.05), and acetate content was significantly elevated in the MIX group relative to CON (*p* < 0.05). Propionate levels did not differ significantly among treatments (*p* > 0.05). At day 42, cecal acetate, propionate, and butyrate concentrations were numerically higher in all supplemented groups (CB, GOD, and MIX) than in the CON group, but these differences did not reach statistical significance (*p* > 0.05).

## 4. Discussion

Oxidative stress arises when reactive oxygen species (ROS) production overwhelms the antioxidant defense system, and the intestine is particularly susceptible to such stress [30,31,32]. Key antioxidant enzymes—including SOD, CAT, and GSH-Px—together with T-AOC are widely used as indicators of antioxidant status, while MDA serves as a marker of lipid peroxidation [33,34,35]. Previous studies have shown that dietary CB can reduce ROS and MDA levels and increase T-AOC and SOD activities in broilers [36,37], whereas GOD has been reported to increase GSH-Px activity, possibly by consuming oxygen during glucose oxidation [38,39,40].

In the present study, however, the antioxidant responses to CB and GOD were not uniform across parameters or growth stages. In the starter phase, only GOD significantly increased ileal SOD activity, whereas CB and MIX showed only numerical increases. In the grower phase, only MIX significantly reduced MDA content, and only CB significantly upregulated GSH-Px activity. These stage-dependent and parameter-specific responses suggest that CB and GOD may act through different mechanisms. CB may enhance antioxidant capacity primarily through butyrate-mediated regulation of redox signaling, whereas GOD may limit ROS production by reducing local oxygen tension. The MIX group showed the most consistent reduction in MDA during the grower phase, which may indicate that the combination provides complementary antioxidant effects under conditions of increasing oxidative challenge. Nevertheless, because the responses were not uniform, these data do not support a broad conclusion of alleviating ROS-mediated oxidative damage; rather, they indicate that CB and GOD modulate specific antioxidant endpoints at specific stages. Further studies measuring direct ROS levels or oxidative damage markers are needed to clarify the functional significance of these changes.

Immunosuppression arising from intensive rearing conditions, stress, and pathogens significantly undermines growth, product quality, and profitability in poultry production [41,42]. The intestinal immune system, being the largest in the body, has been implicated in systemic diseases, as growing data link gut immune responses to extra-intestinal disease processes [43]. Cytokines are small, biologically active proteins synthesized by immune cells that play essential roles in regulating immune responses, inflammation, and tissue repair [44]. Pro-inflammatory and anti-inflammatory cytokines are central to enhancing cellular immunity during inflammation and maintaining immune homeostasis [45]. Immunoglobulins are functional proteins produced by plasma cells, which differentiate from B lymphocytes upon antigen stimulation. They serve as essential effectors in the host’s defense against pathogens [46]. sIgA, a key effector of intestinal mucosal immunity, neutralizes and eliminates invading pathogens such as viruses and bacteria, thereby protecting the intestinal mucosal barrier [47]. A previous study reported that *Clostridium perfringens* infection triggered severe intestinal inflammation in broilers, and the addition of 2 × 10^8^ CFU/g CB effectively mitigated this damage by stimulating the production of anti-inflammatory cytokines such as IL-10 and TGF-β [48]. CB supplementation has also been reported to inhibit small intestinal inflammation and improve intestinal morphology in weaned piglets [49]. The intestinal pH reduction and inhibition of pathogen colonization achieved by GOD-derived GA improve the microbial ecosystem of the gut, thus facilitating immunomodulation [50]. Moreover, dietary GOD has been shown to significantly reduce jejunal mucosal IL-10 and interferon-γ levels in broilers [40]. It has been reported that dietary supplementation with CB significantly elevated jejunal sIgA levels in broilers challenged with enterotoxigenic *Escherichia coli* K88 [51]. It has also been demonstrated that the inclusion of GOD in broiler diets tended to elevate jejunal sIgA concentrations [5].

Our findings reinforce the conclusion that dietary CB and GOD have immunomodulatory effects in broilers. During the starter phase, jejunal IL-2 and IL-6 levels in the MIX group were significantly lower than those in the CON group, by 45.71% and 41.11%, respectively, and the reductions were greater than those observed in the CB and GOD groups. Meanwhile, the jejunal IL-10 level in the MIX group increased by 17.12%, an elevation that also surpassed those in the CB and GOD groups. The GOD and MIX groups exhibited significantly higher jejunal sIgA levels relative to the CON group. At the grower stage, IL-2 levels were markedly lower and IL-10 levels were markedly higher in the MIX group than in the CON group. sIgA levels were not significantly different but tended to increase and remained higher than in the other groups. The combination of CB and GOD exerted a more potent immunomodulatory effect than either agent used alone, as reflected by more sustained elevations in intestinal sIgA. Collectively, our data show that dietary CB and GOD supplementation modulates cytokine balance and curbs excessive inflammation in white-feathered broilers.

Tight junctions (TJs) maintain the selective permeability that characterizes the intestinal epithelial barrier. Disruption of TJs increases the paracellular penetration of luminal macromolecules and harmful substances, triggering mucosal immune responses and inflammation, and potentially leading to intestinal or even systemic diseases [52]. Gut health is largely determined by the permeability of the intestinal barrier, which also constitutes the primary defense against microbial pathogens. This barrier function is closely associated with intestinal oxidative defense, inflammatory responses, and immune status [53,54]. It has been shown that CB enhances intestinal barrier integrity and upregulates the relative expression of the TJ proteins *ZO-1* and *Claudin-1* [55]. Another study demonstrated that CB significantly elevated jejunal *Occludin* and *ZO-1* expression in broilers [56]. The expression of TJ protein genes was enhanced by GOD at 75 U/kg, as documented in earlier investigations [54]. It has been reported that dietary GOD at 200 U/kg elevates the mRNA expression of *ZO-1*, *Claudin-1*, and *Claudin-2* in ducks [57]. These findings indicate that CB and GOD can enhance intestinal mucosal integrity and reduce intestinal permeability by upregulating TJ protein expression. The present data suggest that CB and GOD strengthen intestinal mucosal integrity and lower permeability through increased expression of TJ proteins [58].

Compared with CON, the CB and GOD groups had significantly higher jejunal *Occludin* mRNA expression during the starter phase, whereas the MIX group showed only a numerical increase that did not reach statistical significance (*p* > 0.05). During the grower phase, jejunal *ZO-1* mRNA expression was significantly higher in the MIX group than in the CON group, while the CB and GOD groups exhibited numerical increases without statistical significance (*p* > 0.05). The combination of CB and GOD may have enhanced the intestinal microenvironment by providing GA (from GOD) and butyrate (from CB), thereby reducing the colonization of enteric pathogens and strengthening barrier function, ultimately leading to the upregulation of *ZO-1* expression. These results suggest that both individual and combined supplementation with CB and GOD can modulate TJ protein expression in the jejunum through their respective metabolic pathways, thereby enhancing intestinal mucosal barrier function. The stage-dependent and treatment-specific nature of these transcriptional changes may reflect differences in the mechanisms by which CB and GOD influence TJ-related gene expression. CB is known to produce butyrate, which can serve as an energy substrate for enterocytes and may also regulate gene transcription through histone deacetylase inhibition, thereby potentially affecting the expression of TJ components such as *Occludin* and *ZO-1*. GOD, on the other hand, catalyzes the oxidation of glucose to produce gluconic acid and hydrogen peroxide, thereby lowering the local oxygen tension and modifying the intestinal redox environment, which may indirectly influence epithelial gene expression. The combination of CB and GOD may improve the intestinal microenvironment by providing gluconic acid (from GOD) and butyrate (from CB), which together could create conditions more favorable for the maintenance of epithelial barrier-related gene expression, particularly *ZO-1* during the grower phase.

However, it is important to note that these data represent mRNA expression levels of selected barrier-associated genes and do not directly demonstrate functional changes in barrier permeability or integrity. The absence of consistent effects across all genes and time points suggests that the regulation of TJ expression by CB and GOD is not uniform and may depend on growth stage, local intestinal conditions, and the specific gene examined. Future studies incorporating direct measurements of intestinal permeability, TJ protein abundance, and localization would help to confirm the functional relevance of these transcriptional changes. Overall, these results indicate that both individual and combined supplementation with CB and GOD can modulate the mRNA expression of TJ proteins in the jejunum, but the functional implications of these changes require further investigation.

The cecum is the primary site in broilers where the gut microbiota ferments indigestible dietary carbohydrates, with SCFAs as the main metabolic products. SCFAs—chiefly acetate, propionate, and butyrate—make up over 95% of the total. Butyrate serves as the principal energy source for gut epithelial cells, while acetate mediates cross-feeding among gut microbes, and propionate is chiefly concerned with host metabolic regulation of glucose and lipids [59]. SCFAs are critical for maintaining broiler intestinal health, largely through their influence on TJ integrity [60,61]. It has been reported that early oral administration of CB (2.0 × 10^9^ CFU/mL) to Muscovy ducks for three consecutive days alters gut microbial composition and elevates cecal acetate, propionate, and butyrate concentrations [28]. Previous work has shown that CB supplementation in Pekin ducks not only improves growth, immunity, SCFAs, and TJ protein expression but also elevates the richness and diversity of the gut microbiota [62]. GA, produced from GOD-catalyzed reactions in the gut, serves to reduce intestinal pH and boost digestive enzyme activity, thereby promoting animal growth as a key organic acid [63]. It has been shown that GA promotes butyrate production in batch cultures of porcine cecal digesta [64]. GA supplementation improved piglet growth performance, an effect that may be linked to its capacity to raise total SCFAs—especially butyrate—as demonstrated in in vitro incubations with porcine cecal inoculum [65].

During the starter phase, although the GOD and MIX groups exhibited only non-significant increases, the CB group showed a statistically significant rise of 91.57% in cecal butyrate versus CON, consistent with earlier observations. In the MIX group, cecal acetate content increased significantly by 66.07% relative to CON. In contrast, the CB and GOD groups showed only numerical increases that did not reach statistical significance (*p* > 0.05). The highest butyrate concentration was observed in the CB group, which may be directly attributable to the butyrate-producing capacity of CB. The MIX group had a lower butyrate level than the CB group but displayed a significant increase in acetate. Although acetate and propionate in the MIX group did not differ significantly from CON at the grower phase, they were numerically higher than the corresponding levels in the other groups. These findings suggest that both CB and GOD, whether administered singly or together, can influence cecal SCFA profiles. However, the microbial mechanisms underlying these changes remain to be determined, because microbial composition and abundance were not assessed in this study. In particular, the increase in acetate observed with combined supplementation may reflect a more favorable intestinal environment for acetate-producing bacteria, but this possibility requires confirmation through future studies combining SCFA profiling with 16S rRNA sequencing or metagenomic approaches.

The present study has several limitations. First, intestinal microbiota composition was not analyzed; therefore, the microbial mechanisms proposed for the SCFA changes remain speculative. Second, intestinal barrier function was assessed only at the mRNA level, and direct measurements of TJ protein abundance, permeability, or histological integrity were not performed. Third, growth performance parameters were not reported, which limits the practical implications of the findings. Fourth, the total additive dose in the MIX group was 800 mg/kg, whereas each single-additive group received 400 mg/kg; therefore, it is not possible to distinguish whether the superior effects observed in the MIX group were attributable to the higher total dose or to the simultaneous use of the two additives. If the underlying mechanisms are to be clarified in future studies, a dose-matched group should be included. Fifth, statistical comparisons among treatment groups were performed using one-way ANOVA without formal interaction testing; therefore, the present results cannot establish synergistic effects between CB and GOD. Future studies should address these limitations by incorporating microbiome analysis, functional barrier assessments, growth performance measurements, dose–response designs, and factorial interaction testing.

## 5. Conclusions

In conclusion, combined supplementation with CB and GOD was able to modulate cecal SCFA profiles, antioxidant enzyme activities, immune-related parameters, and the mRNA expression of intestinal barrier-associated genes in broilers, with effects varying by parameter and growth stage. These results suggest that the CB–GOD combination has the potential to serve as a dietary strategy for supporting intestinal health in broilers, and provide a theoretical basis and operational reference for the rational combination of probiotics and enzymes in broiler diets.

Under the present experimental conditions, the combined supplementation group showed superior effects compared with the individual supplementation groups; however, this superiority may not arise from the combination effect per se, but may merely be related to the increase in total additive dose. Future studies are warranted to include a dose-matched group to further verify the independent contribution of the combination effect.

## Figures and Tables

**Table 1:** Formulation and nutrient levels of the basal diet (%, as-fed basis).

Items	1–21 d	22–42 d
Ingredients		
Corn	58.95	60.9
Soybean oil	3	3
Soybean meal	23	20
Cottonseed cake	6	6
Rape seed cake	5	6
Limestone	1.07	1.22
CaHPO_4_	1.9	1.8
NaCl	0.37	0.37
Choline	0.11	0.11
Vitamin premix ^1^	0.1	0.1
Mineral premix ^2^	0.5	0.5
Total	100	100
Nutrient levels		
ME (MJ/kg)	12.26	12.64
CP (%)	21.00	20.00
CF (%)	7.00	6.00
Ca (%)	1.05	0.95
P (%)	0.52	0.47
Met + Cys (%)	0.82	0.71

Note: ^1^ The vitamin premix provides the following per kg of the diet: VA—9000 IU; VB1—3.00 mg, VB_2_—8.00 mg, VB_6_—4.40 mg, VB_12_—0.012 mg, VD_3_—3000 IU, VE—26 IU, VK_3_—1.20 mg, calcium pantothenate—1 mg, nicotinic acid—45 mg.; ^2^ The mineral premix provides the following elements per kg of the diet: Fe—100 mg, Cu—10 mg, Mn—120 mg, Zn—108 mg, I—1.50 mg, Se—0.35 mg. ME is calculated, and the rest are measured. Abbreviations: ME: metabolic energy; CP: crude protein; CF: crude fiber.

**Table 2:** Primer sequences used for quantitative real-time PCR.

Gene	Sequence (5′–3′)	GenBank Accession No.
*β-* *actin*	F: 5-GCCAACAGAGAGAAGATGACAC-3 R: 5-GTAACACCATCACCAGAGTCCA-3	NM_205518
*Occludin*	F: 5-GAGCCCAGACTACCAAAGCAA-3 R: 5-GCTTGATGTGGAAGAGCTTGTTG-3	NM_205128.1
*ZO-1*	F: 5-CCGCAGTCGTTCACGATCT-3 R: 5-GGAGAATGTCTGGAATGGTCTGA-3	XM_040680630.2
*JAM2*	F: 5-AGCCTCAAATGGGATTGGATT-3 R: 5-CATCAACTTGCATTCGCTTCA-3	XM_046907880.1
*MUC2*	F: 5-GCCTGCCCAGGAAATCAAG-3 R: 5-CGACAAGTTTGCTGGCACAT-3	XM_046942297.1

Abbreviations: F = forward primer, R = reverse primer. Zonula occludens protein 1 (ZO-1), junctional adhesion molecule 2 (JAM2), mucin 2 (MUC2).

**Table 3:** Effects of CB and GOD on ileal antioxidant capacity in broilers.

Items	Groups				SEM	*p*-Value
	CON	CB	GOD	MIX		
21 d						
CAT (U/mg prot.)	2.42	2.60	2.64	2.98	0.816	0.799
GSH-Px (U/mg prot.)	19.78	22.16	23.00	21.66	0.802	0.626
SOD (U/mg prot.)	24.07 ^a^	26.45 ^ab^	28.95 ^b^	26.07 ^ab^	0.605	0.034
T-AOC (mmol/mg prot.)	0.77	0.84	0.86	0.84	0.016	0.221
MDA (nmol/mg prot.)	1.03	0.92	0.96	0.75	0.051	0.237
42 d						
CAT (U/mg prot.)	3.75	4.15	4.20	4.91	1.096	0.415
GSH-Px (U/mg prot.)	23.85 ^a^	30.10 ^b^	27.80 ^ab^	25.27 ^a^	0.681	0.002
SOD (U/mg prot.)	21.27	24.45	23.02	24.05	0.532	0.143
T-AOC (mmol/mg prot.)	0.84	0.97	0.89	0.88	0.018	0.071
MDA (nmol/mg prot.)	0.89 ^a^	0.78 ^ab^	0.82 ^ab^	0.65 ^b^	0.031	0.032

Note: CON = fed basal diet; CB = fed basal diet + 400 mg/kg CB; GOD = fed basal diet + 400 mg/kg GOD; MIX = fed basal diet + 400 mg/kg CB + 400 mg/kg GOD. Within a row, means bearing different superscript letters differ significantly (*p* < 0.05). SEM, standard error of the mean (*n* = 6).

**Table 4:** Effects of CB and GOD on jejunal immune function in broilers.

Items	Groups				SEM	*p*-Value
	CON	CB	GOD	MIX		
21 d						
IL-1β (pg/mg prot.)	18.68	13.14	18.48	16.76	1.185	0.407
IL-2 (pg/mg prot.)	16.99 ^a^	13.84 ^ab^	15.32 ^ab^	11.66 ^b^	0.603	0.009
IL-6 (pg/mg prot.)	17.24 ^a^	13.56 ^b^	15.05 ^ab^	12.60 ^b^	0.501	0.005
IL-10 (pg/mg prot.)	16.94 ^b^	18.39 ^ab^	17.33 ^ab^	20.44 ^a^	0.475	0.048
TNF-α (pg/mg prot.)	11.23	10.81	9.50	8.78	0.403	0.128
sIgA (pg/mg prot.)	10.50 ^a^	15.86 ^ab^	20.79 ^b^	20.75 ^b^	1.397	0.012
42 d						
IL-1β (pg/mg prot.)	18.78	16.95	15.08	14.85	1.656	0.199
IL-2 (pg/mg prot.)	17.86	15.02	17.08	15.22	0.592	0.231
IL-6 (pg/mg prot.)	17.51 ^a^	13.62 ^ab^	16.13 ^ab^	12.91 ^b^	0.642	0.030
IL-10 (pg/mg prot.)	16.73 ^a^	19.00 ^ab^	18.87 ^ab^	20.26 ^b^	0.463	0.039
TNF-α (pg/mg prot.)	8.50	7.43	5.90	5.81	0.613	0.359
sIgA (pg/mg prot.)	13.65	14.35	14.30	16.46	0.526	0.322

Note: CON = fed basal diet; CB = fed basal diet + 400 mg/kg CB; GOD = fed basal diet + 400 mg/kg GOD; MIX = fed basal diet + 400 mg/kg CB + 400 mg/kg GOD. Within a row, means bearing different superscript letters differ significantly (*p* < 0.05). SEM, standard error of the mean (*n* = 6).

**Table 5:** Effects of CB and GOD on intestinal barrier function in broilers.

Items	Groups				SEM	*p*-Value
	CON	CB	GOD	MIX		
21 d						
*JAM2*	1.00	1.14	1.42	1.25	0.074	0.239
*MUC2*	1.00	1.14	1.02	1.06	0.045	0.728
*Occludin*	1.00 ^a^	1.97 ^c^	1.58 ^bc^	1.32 ^ac^	0.118	0.008
*ZO-1*	1.00	1.30	1.11	1.03	0.071	0.493
42 d						
*JAM2*	1.00	1.17	1.49	1.29	0.088	0.234
*MUC2*	1.00	1.20	1.09	1.18	0.079	0.814
*Occludin*	1.00	1.20	1.45	1.31	0.077	0.169
*ZO-1*	1.00 ^a^	1.04 ^a^	1.28 ^ab^	1.54 ^b^	0.074	0.025

Note: CON = fed basal diet; CB = fed basal diet + 400 mg/kg CB; GOD = fed basal diet + 400 mg/kg GOD; MIX = fed basal diet + 400 mg/kg CB + 400 mg/kg GOD. Within a row, means bearing different superscript letters differ significantly (*p* < 0.05). SEM, standard error of the mean (*n* = 6).

**Table 6:** Effects of CB and GOD on cecal SCFA levels in broilers.

Items	Groups				SEM	*p*-Value
	CON	CB	GOD	MIX		
21 d						
Acetate (mmol/mL)	5.04 ^a^	6.67 ^ab^	7.96 ^ab^	8.37 ^b^	0.472	0.049
Propionate (mmol/mL)	0.97	1.04	1.44	1.03	0.105	0.458
Butyrate (mmol/mL)	0.83 ^a^	1.59 ^b^	0.96 ^ab^	1.39 ^ab^	0.107	0.023
42 d						
Acetate (mmol/mL)	8.72	11.50	10.81	16.72	1.087	0.234
Propionate (mmol/mL)	1.13	1.64	1.38	2.07	0.160	0.255
Butyrate (mmol/mL)	1.1	1.63	1.44	1.44	0.068	0.134

Note: CON = fed basal diet; CB = fed basal diet + 400 mg/kg CB; GOD = fed basal diet + 400 mg/kg GOD; MIX = fed basal diet + 400 mg/kg CB + 400 mg/kg GOD. Within a row, means bearing different superscript letters differ significantly (*p* < 0.05). SEM, standard error of the mean (*n* = 6).

## Data Availability

The original contributions presented in this study are included in the article.
